# Spiking Neural Networks Based on OxRAM Synapses for Real-Time Unsupervised Spike Sorting

**DOI:** 10.3389/fnins.2016.00474

**Published:** 2016-11-03

**Authors:** Thilo Werner, Elisa Vianello, Olivier Bichler, Daniele Garbin, Daniel Cattaert, Blaise Yvert, Barbara De Salvo, Luca Perniola

**Affiliations:** ^1^Laboratoire d'Électronique et de Technologie de l'Information (LETI), Commissariat à l'Énergie Atomique et aux Énergies Alternatives (CEA)Grenoble, France; ^2^Université Grenoble AlpesGrenoble, France; ^3^Laboratoire d'Intégration de Systèmes et de Technologies (LIST), Commissariat à l'Énergie Atomique et aux Énergies Alternatives (CEA)Gif-sur-Yvette, France; ^4^Institut de Neurosciences Cognitives et Intégratives d'Aquitaine, Université de Bordeaux, CNRSBordeaux, France; ^5^BrainTech Laboratory U1205, Institut National de la Santé et de la Recherche MédicaleGrenoble, France; ^6^BrainTech Laboratory U1205, Université Grenoble AlpesGrenoble, France

**Keywords:** brain-computer interfaces, neuromorphic computing, OxRAM, resistive RAM (RRAM) synapse, spike sorting, spiking neural network, spike timing-dependent plasticity

## Abstract

In this paper, we present an alternative approach to perform spike sorting of complex brain signals based on spiking neural networks (SNN). The proposed architecture is suitable for hardware implementation by using resistive random access memory (RRAM) technology for the implementation of synapses whose low latency (<1μs) enables real-time spike sorting. This offers promising advantages to conventional spike sorting techniques for brain-computer interfaces (BCI) and neural prosthesis applications. Moreover, the ultra-low power consumption of the RRAM synapses of the spiking neural network (nW range) may enable the design of autonomous implantable devices for rehabilitation purposes. We demonstrate an original methodology to use Oxide based RRAM (OxRAM) as easy to program and low energy (<75 pJ) synapses. Synaptic weights are modulated through the application of an online learning strategy inspired by biological Spike Timing Dependent Plasticity. Real spiking data have been recorded both intra- and extracellularly from an *in-vitro* preparation of the Crayfish sensory-motor system and used for validation of the proposed OxRAM based SNN. This artificial SNN is able to identify, learn, recognize and distinguish between different spike shapes in the input signal with a recognition rate about 90% without any supervision.

## 1. Introduction

**Figure 1:**
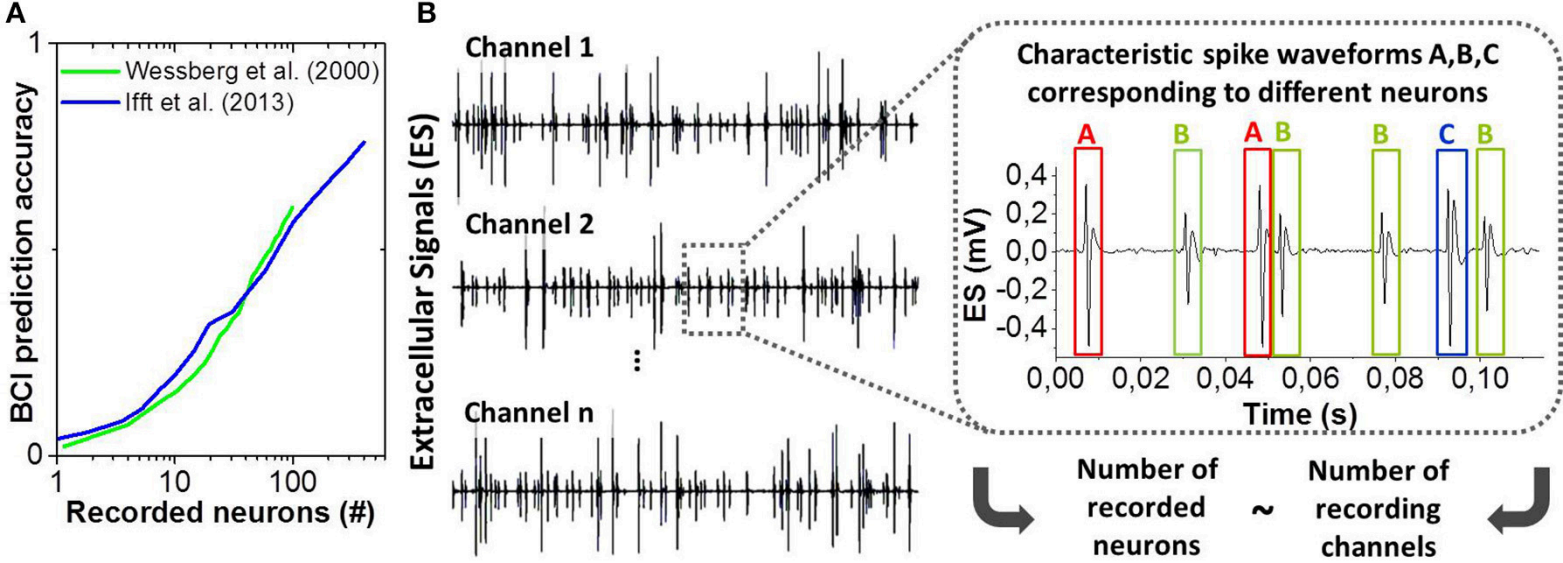
**(A)** Accuracy of BCI or neural prosthesis applications as a function of the number of recorded neurons. Adapted from Wessberg et al. (2000) and Ifft et al. (2013). **(B)** Example of extracellular electrical signals recorded from *n* channels (microelectrodes) and zoom-in showing three distinct spike shapes (A, B, and C) corresponding to three different neurons.

Probing motor cortical activity has recently received increased attention for the exploitation of human brain signals within Brain-Computer Interfaces (BCI). BCI's offer promising rehabilitation approaches to improve life quality of patients suffering from neurodegenerative diseases or paralysis (Hochberg et al., 2006, 2012). This requires the ability to precisely collect and analyse brain signals, e.g., triggered when a person intends to perform movements. The effectiveness and accuracy of BCI systems scale with the number of simultaneously recorded populations of neurons (see Figure 1A) (Wessberg et al., 2000; Ifft et al., 2013). Advanced microelectrode array (MEA) technologies (Spira and Hai, 2013) are unique and increasingly powerful tools to explore the central nervous system in detail. Nowadays, they consist of hundreds or thousands of microelectrodes that allow recording the activity of large neural ensembles and especially spikes (action potentials) generated by the surrounding single cells (see Figure 1B). These technologies generate massive data due to sampling rates of typically 20–40 kHz that have to be processed for further use and/or wireless transmission (Yin et al., 2014). Spike sorting is a key technique to drastically reduce the amount of data by extracting relevant information as how many cells are active and the different instants at which they fire (Abeles and Goldstein, 1977). State-of-the-art spike sorting methodologies are mainly based on spike shape template matching and statistical clustering techniques (Lewicki, 1998; Rey et al., 2015), where the electrical waveform is analyzed against a temporally sliding analysis window and a spike is identified whenever the waveform is found to match one of the previously generated templates or certain set of parameters within some tolerance. The most commonly used spike sorting approach consists of spike detection [mainly by thresholding (Tanskanen et al., 2015)], feature extraction (typically Principal Components Analysis, PCA) and clustering (e.g., k-means). Algorithms of this type have been implemented in commercial software (Bestel et al., 2012), however, they present several limitations, as they often need user supervision (manual tuning of the threshold parameters, choice of features to be extracted), they can fail to recognize overlapping spikes and moreover they are computationally expensive. Therefore, most of the neural signal processing is performed via offline software on desktop computers. The offline processing is not optimal because it does not allow for real-time processing in closed-loop applications [e.g., in BCI (Hochberg et al., 2006, 2012)] or real-time data compression prior to wireless transmission with reasonable power consumption in case of high channel counts. It was shown in Wessberg et al. (2000) and Ifft et al. (2013) that BCI performances are enhanced when recording from large numbers of neurons by means of large MEA's, i.e., numerous signals have to be stored and decoded resulting in exploding data rates and computational efforts, respectively. Furthermore, the offline processing using computers or powerful GPU's is an issue for the design of power-efficient portable BCI solutions. New spike sorting approaches are required to address the described drawbacks of state-of-the-art techniques.

In this paper, we explore the design of an RRAM based neuromorphic system targeting to perform real-time spike sorting with nanowatt-level power consumption and reasonable spike sorting performances. Brain-inspired computing imitations by means of neuromorphic network architectures have demonstrated to be superior candidates for the detection and prediction of patterns occuring in complex data with respect to conventional von-Neumann architectures (Ananthanarayanan et al., 2009; Merolla et al., 2014; Prezioso et al., 2015). Furthermore, emerging resistive RAM (RRAM) memories offer the possibility to build complex brain-like cognitive computing systems that are compact and consume low power. Several concepts for synaptic implementations based on RRAM have been proposed (Wu et al., 2012; Kuzum et al., 2013). Oxide based RRAM (OxRAM) technology is among the most promising candidates for next generation Non Volatile Memories (NVM) thanks to its low (sub-μ*A*) operation currents (Goux et al., 2012), highly scalable lateral dimensions (Govoreanu and Kar, 2011), low cost production, and back-end-of-line (BEOL) process compatibility. While OxRAM in typical NVM applications is operated using switching currents higher than 50 μ*A* for reliability reasons, we have analyzed the OxRAM device behavior in this paper for switching currents as low as 1 μ*A*. Switching and conduction properties are investigated in the perspective of implementation into potential artificial synapses for neuromorphic systems.

This paper is structured as follows. Section 2 introduces the biological data used in this paper illustrating the spike sorting problem. Section 3 describes the architecture of the SNN, followed by the electrical characterization of OxRAM and its implementation into an artificial synapse in Sections 4 and 5, respectively. Section 6 presents the performance of the spike sorting application and finally, Section 7 summarizes our findings.

## 2. Biological data

**Figure 2:**
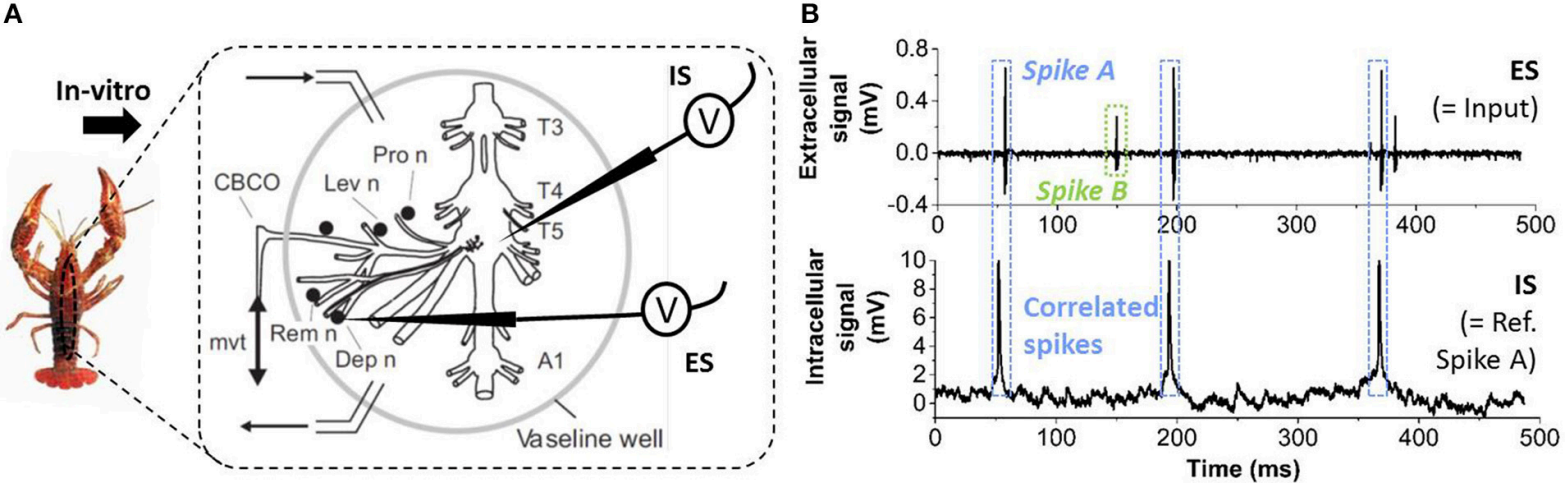
**(A)** Illustration of the experiment used to obtain real biological data. The crayfish is dissected and two electrodes are used *in-vitro*, one intracellular electrode inside a motor neuron in the T5 ganglion and one extracellular positioned against a depressor nerve (“Dep”). **(B)** The extracellular signal (ES, short sequence shown) contains two different spike shapes, labeled as Spike A, B. The intracellular signal (IS) contains spiking events matching only Spike A of the ES.

In order to illustrate the validity of the proposed spike sorting methodology, we measured the extracellular activity from *in-vitro* Crayfish nerves recorded simultaneously with intracellular data of one motor or sensory neuron of the T5 ganglion (see Figure 2A) (Cattaert and Manira, 1999; Cattaert et al., 2010). In these data, the extracellular signal (ES) contains two different spike shapes (labeled as Spike A and B in Figure 2B) corresponding to two different neurons. The spikes simultaneously observed in the intracellular signal (IS) correlate with the activity of Spike A in the ES. Therefore, the IS activity can be used as the ground truth to assess the spike sorting capability of our system for the detection of Spike A in the ES data. The entire data set duration comprises 681 s and is called CF1 subsequently.

## 3. Spiking neural network (SNN) architecture for spike sorting

**Figure 3:**
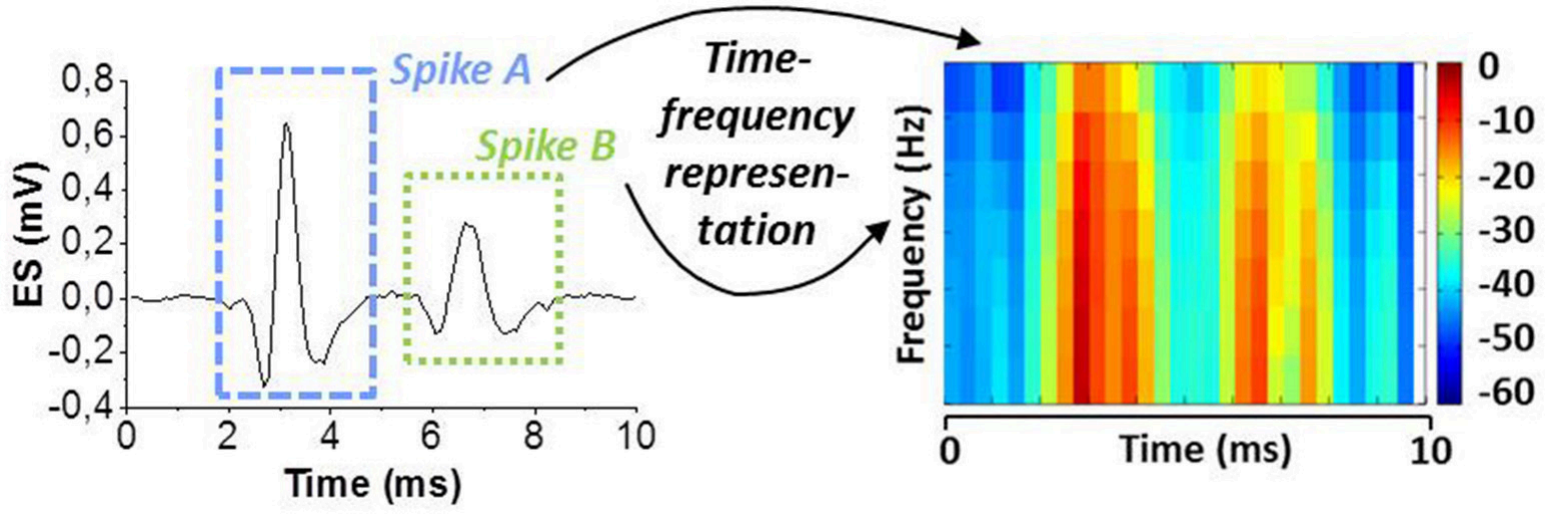
**Spike sorting paradigm based on continuous time-frequency decomposition of the analog extracellular signal (ES)**. Different spike shapes (here Spike A and B) exhibit distinct patterns in the spectrogram. This “finger print” is used for spike shape recognition.

**Figure 4:**
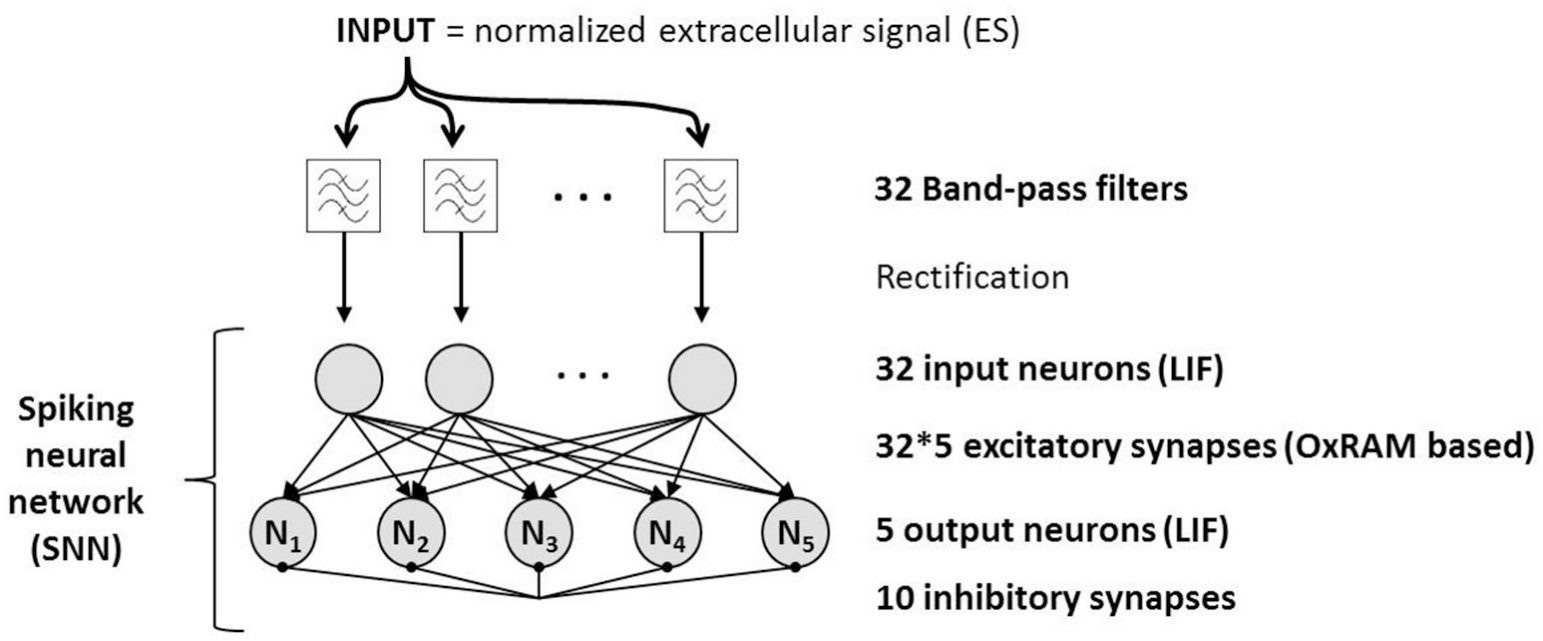
**Functional schematic of spike sorting system based on a Spiking Neural Network**. The extracellular signal (ES) is fed through 32 frequency band-pass filters which are connected one-to-one to the input layer of the SNN. Synapses are based on OxRAM devices. Output neurons are interconnected by inhibitory synapses to feature the winner-take-all principle which allows them to become selective to different input spikes shapes.

We assume that different spike shapes observed in the extracellular signal exhibits distinct representations in the time-frequency domain as shown for example in Figure 3 for “Spike A” and “Spike B” which can serve as finger prints for the identification of these spike shapes. By this approach, it is possible to trace the activity of single neurons. Figure 4 shows the topological view of the two-layer SNN architecture (implemented in the event-driven simulator “Xnet”(Bichler et al., 2013) designed to extract, learn, and recognize different spike shapes from biological extracellular signals. The topmost layer above the SNN represents the frequency band-pass filtering used to convert the extracellular recording into a useful input for the SNN. Thus, the normalized continuous ES is encoded by 32 frequency band-pass filters whereas their center frequencies are gradually increasing with the filter number. The 32 filtered signals are then full-wave rectified and presented to the SNN input layer of 32 neurons where the analog continuous signals are converted into spikes which are then propagated along the synapses to the SNN output layer of 5 neurons. The neurons of both layers are described by the Leaky Integrate Fire (LIF) model (Gerstner and Kistler, 2002) and they are fully connected by 32 × 5 excitatory synapses, i.e., every input neuron has a synaptic connection with every output neuron. The firing event of an output neuron indicates that the spike inspected in the input signal (Spike A or B in the example of Figure 3) belongs to the specific class corresponding to this output neuron.

The parameters for the band-pass filters and input neurons are tuned separately from the parameters of the output neurons. First, the order and the bandwidth of the filters have to be defined. Note, that spikes in the recorded extracellular signal have a characteristic duration of 1–2 ms and multiple spikes can occur within few tens of milliseconds. Hence, the filter signals must allow to resolve and distinguish those different spike signals sufficiently. Moreover, our spike sorting approach aims at real-time application in BCI which requires minimized delay times between input (spike event in the extracellular signal) and output (corresponding output neuron of SNN spikes). To address this need, a low filter order (≤3) is required in order to have filter responses of less than a few ms. Filter bandwidths should be narrow to achieve a reasonable frequency resolution, however, the temporal resolution degrades (longer filter response) as the filter bandwidth is reduced. Thus, a trade-off between frequency and temporal resolution has to be found. Bandwidths of around 60 Hz and 2nd order Butterworth filters offer a good compromise for our application. The frequency spectrum of spikes is usually invariant and does not exceed 3000 Hz (Rey et al., 2015; Kellis et al., 2016). In this work, we defined a frequency range of 100–2000 Hz for the signal analysis which allows to exclude low frequent background signals (below 100 Hz). Finally, the number of filters depends on the previously defined filter bandwidth and on the frequency range (100–2000 Hz) to be analyzed. It is chosen such that the entire frequency range is covered without introducing excessive filter redundancy among the adjacent filters. We have used 32 band-pass filters which are distributed on a linear range between 100 and 2000 Hz. This filter bank is independent of the specific dataset chosen for this work and can be used on different spiking neural data as demonstrated in Section 6.

By using the band-pass filter approach to encode spiking data, the SNN does receive strong input signals if a spike is observed in the input data whereas rather low-frequency signals are not able to excite the network sufficiently. Thus, no dedicated method to remove low frequent noise is required and spike detection is inherently implemented. As shown in Figure 4, the number of input neurons corresponds to the number of filters. The corresponding LIF parameters are manually tuned using the two spike waveforms of the biological dataset described in Section 2. The parameters are tuned in such a way that the 1st layer's activity represents the spectral magnitude of the signal throughout the tested frequency range (100 Hz–2000 Hz), i.e., the stronger the energy in a specific frequency band the more input spikes are generated. Thus, the input neurons create characteristic patterns for different spike waveforms.

The number of output neurons determines the maximum number of spike classes that the SNN is able to classify. A sufficiently high number of output neurons has to be chosen so that every spike shape contained in the extracellular data can be assigned to one output neuron, i.e., the number of output neurons has to be at least as high as the (a priori unknown) number of spike shapes in the extracellular signal. Here, our dataset of Section 2 contains two spike classes which need to be classified. However, the number of classes is typically not known in this kind of application, therefore we used five output neurons to verify that our network is able to detect the number of classes independently. The goal is that every spike shape will be learned and recognized by one of the output neurons whereas non-selective neurons remain silent, i.e., the number of spiking output neurons indicates the number of spike classes. To avoid classification redundancy, lateral inhibition is implemented with recurrent inhibitory synapses across the output layer to prevent the neurons from simultaneous spiking (i.e., winner-takes-all principle). The output neuron parameters (I_thres_, T_leak_, T_refractory_) were tuned manually and then optimized by using a genetic algorithm to make the system sensitive for spiking data. In the genetic algorithm, we randomly varied the parameters (maximum 20%) of one generation and evaluated the classification rate. Based on the results of each generation, four winners were chosen for further parameter variation. The level of variation was decreased as the classification rate saturated. The parameters of the LIF input and output neurons are given in Table 1.

**Table 1:** **Leaky Integrate Fire (LIF) neuron parameters of the 2-layer spiking neural network used for spike sorting of extracellular spiking data**.

**Symbol**	**Parameter**	**Layer 1**	**Layer 2**
I_thres_	Integration threshold	0.1 (a.u.)	0.58 (a.u.)
T_leak_	Leak time constant	0.2 ms	5.1 ms
T_refractory_	Refractory period	4 ms	46.1 ms

**Figure 5:**
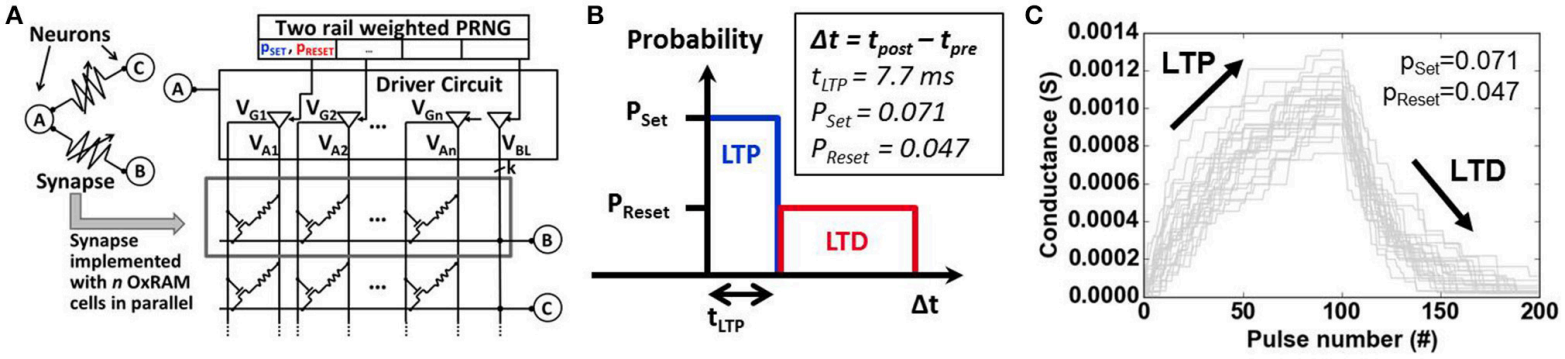
**(A)** Schematic representation of synapses based on binary OxRAM devices. A synapse consists of *n* devices (row) in parallel. A pseudo-random number generator (PRNG) is integrated for probabilistic device programming. **(B)** Probabilistic learning rule used for online learning in our SNN inspired by spike timing dependent plasticity (STDP). Set and Reset probabilities, p_Set_ and p_Reset_, as well as the LTP time window (t_LTP_) are indicated. **(C)** Long Term Potentiation (LTP) and Long Term Depression (LTD) for 20 synapses each based on 20 OxRAM devices using p_Set_ and p_Reset_. OxRAM devices are fitted using experimental data from Figure 9.

One of the key challenges for spike sorting algorithms is the real-time functionality for a priori unknown data. This requires an online learning algorithm, i.e., the fast adaptation of the spike sorting system to new data (new spike shapes in the ES, changing number of classes) and specifically for SNN a synaptic latency that is lower than the duration of biological spikes (approximately 1 *ms*). Spike-timing dependent plasticity (STDP) is used to meet the first requirement whereas the latter is accomplished thanks to the fast switching synapses (<1μs), in our case the OxRAM devices. Note, that a fast switching time of the SNN synapses is required since the online learning is permanently active. Without online learning, classification does not require fast switching synapses. Our synapses are composed of multiple binary-state devices (Figure 5A) in order to achieve multi-level synaptic weights (Bill and Legenstein, 2014). The STDP rule for online learning was simplified and used in a probabilistic manner (Goldberg et al., 2001) (see Figure 5B) to induce gradual Long Term Potentiation (LTP) and Depression (LTD) (Figure 5C). The synaptic weight changes when a post synaptic spike occurs. If the presynaptic neuron was activated recently (Δ*t* < *t*_*LTP*_), LTP is performed on the synapse with a given Set probability *p*_*Set*_, otherwise (Δ*t* > *t*_*LTP*_), LTD is performed with a Reset probability *p*_*Reset*_. The probabilities as well as *t*_*LTP*_ were optimized by means of a genetic algorithm together with the parameters of the output neuron layer. Note that once all the parameters for the filters, SNN and probabilistic STDP are set, the spike sorting system may in principal be used on any spiking dataset without changing those parameters.

It is possible to implement the presented SNN in a co-integrated circuit using complementary metal oxide semiconductor (CMOS) technology for the neurons (Joubert et al., 2012) as well as the band-pass filters and Oxide based resistive RAM (OxRAM) for the synapses (Garbin et al., 2015). The electrical conductance of OxRAM devices can be modified by means of voltage pulses which is exploited to tune the synaptic weights, described in Section4. The synapse design is explained in more detail in Section 5. The validity of the proposed network and the OxRAM synapse model extracted from electrical data will be demonstrated in Section 6 by means of simulations using our special purpose event-driven simulator tool “Xnet.”

## 4. OxRAM electrical device analysis

OxRAM technology relies on a functional thin oxide between two metal layers [the Top (TE) and Bottom electrodes (BE), respectively]. Binary metal oxides were reported to exhibit a sudden switching phenomenon for a critical electric field applied across TE to BE resulting in a drop of electrical resistance of the oxide (Gibbons and Beadle, 1964) leading to the so-called Low Resistance State (LRS). The resistance change is commonly attributed to the formation of an oxygen vacancy (V_O_)-rich path, the so-called Conductive Filament (CF) (Wong et al., 2012). The transformation is partly reversible by breaking the CF when V_O_ are recombining with diffusing oxygen ions, thus leading to the High Resistance State (HRS). Hence, OxRAM offers two distinct non-volatile states, LRS and HRS, when it is operated using fixed programming conditions for Set and Reset. The LRS level depends on the used Set current (I_CC_, also known as current compliance) whereas the HRS level is determined by the applied Reset voltage (V_R_) (Wong et al., 2012). OxRAM suffers from cycle-to-cylce as well as device-to-device variability in both LRS and HRS. This is a major concern for standard non-volatile memory applications, however, neuromorphic applications are expected to be more robust to those single-unit variabilities (Vianello et al., 2015).

**Figure 6:**
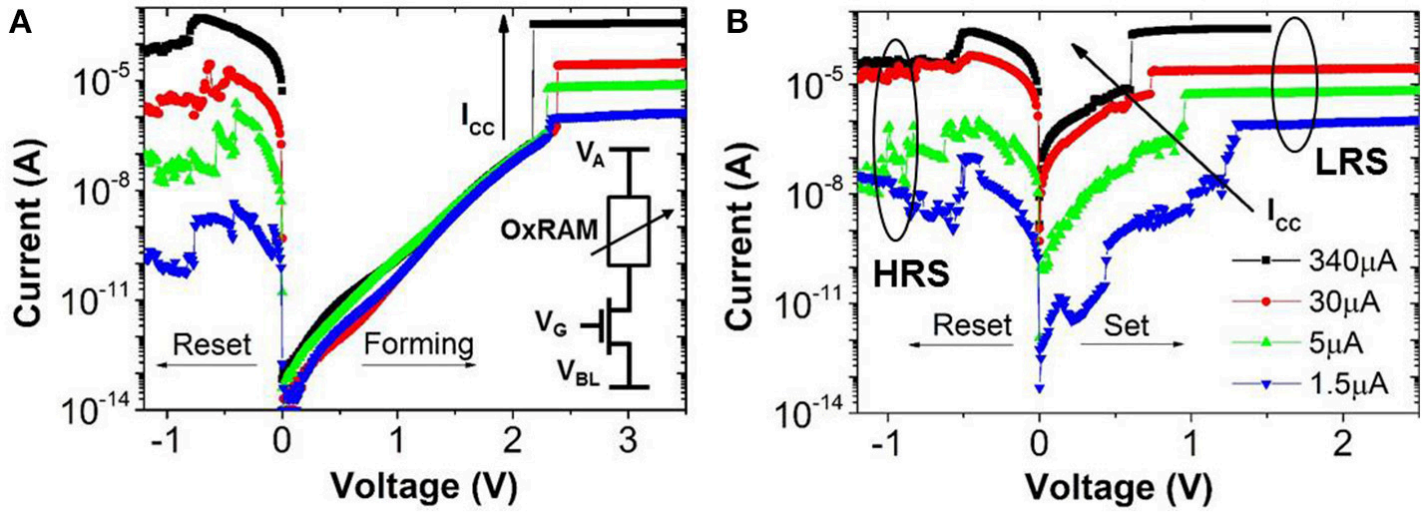
**OxRAM (Al_**2**_O_**3**_/HfO_**2**_) IV characteristics for (A) Forming/1st Reset and (B) Set/Reset**. Operation is shown for I_CC_ (i.e., current compliance) ranging from 1.5 μ*A* to 340 μ*A*. Note the shift of the Set IV curve toward higher voltages for reduced I_CC_.

**Figure 7:**
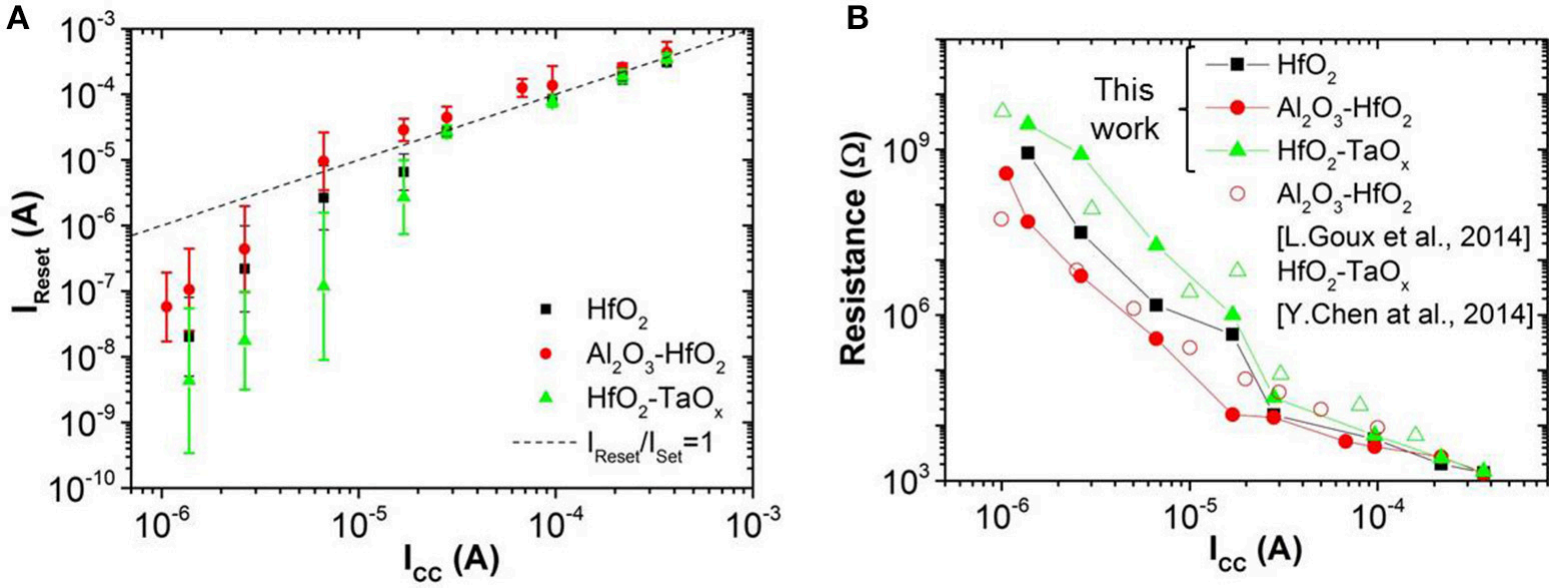
**(A)** Reset current (I_Reset_) as a function of I_CC_ for different oxide materials and **(B)** LRS depending on I_CC_ for different oxide materials.

**Figure 8:**
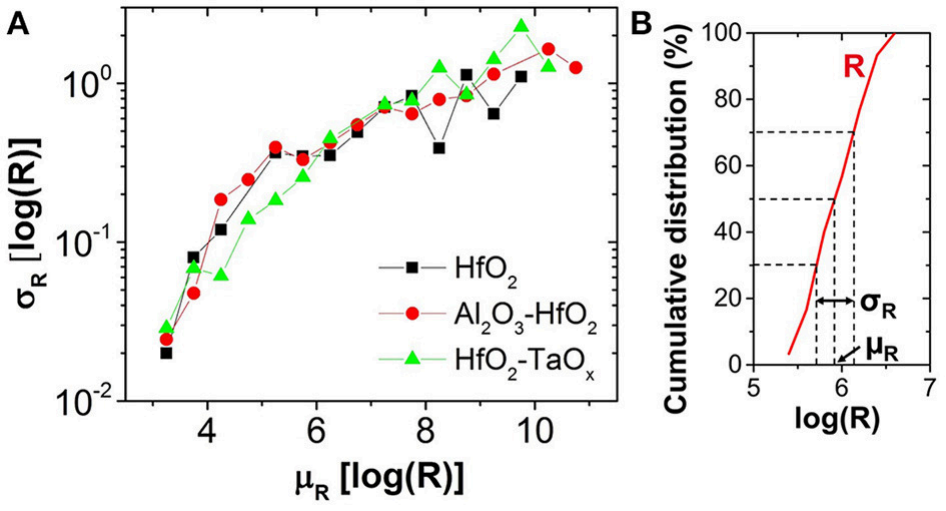
**(A)** Variability (σ_R_) as a function of programmed mean resistance (μ_R_). **(B)** μ_R_ and σ_R_ extraction methodology from experimental resistance distribution of 30 cycles for one device.

In this work, OxRAM resistors are co-integrated with n-type metal oxide semiconductor (NMOS) transistor access devices in a standard 65 nm CMOS technology (Vianello et al., 2014), allowing for a precise current control. The resistive switching layer is sandwiched between 10 nm thick Ti and 35 nm TiN electrodes. Three oxide compositions deposited by Atomic Layer Deposition (ALD) were studied: (i) 5 nm HfO_2_, (ii) 1 nm Al_2_O_3_/3 nm HfO_2_, and (iii) 5 nm HfO_2_/4 nm TaO_x_. To study the electrical behavior of OxRAM with regard to a synapse implementation, significantly lower currents (down to approximately 1 μA) with respect to our previous studies were investigated (>50 μA). Several OxRAM devices were therefore tested both by voltage sweeping (dc) and voltage pulses (ac). All resistance readings of the single OxRAM devices were performed using a bias voltage V_A_ = 0.1 V while reading the static current. Figures 6A,B show typical IV sweep curves for I_CC_ ranging from 1.5 μA to 340 μA for Forming (first Set operation), Set, and Reset operations. During Forming or Set operations, a positive bias voltage is applied to TE to switch the OxRAM devices from HRS to LRS. During Reset operations, a negative bias voltage is applied to TE switching from LRS to HRS. Although the forming voltages (i.e., voltage of abrupt current increase) are similar for all operation currents, the Set voltage increases when I_CC_ is reduced and the Set process appears to be more gradual. Furthermore, the reset current (I_Reset_), defined as the maximum current during the reset process, is typically equal or slightly higher than the current compliance during Set operation. This is true for I_CC_ >20 μA, however, if I_CC_ is reduced below 20 μA, I_Reset_ drops significantly below I_Set_ (Figure 7A). This applies regardless of the oxide material whereas the effect is the strongest for the HfO_2_/TaO_x_ layer which is the oxide layer with the highest overall thickness of 9 nm tested in this work. This suggests that the electric conduction involves mainly tunneling transport phenomena. Figure 7B represents the LRS values as a function of I_CC_ for the different material compositions. While the LRS seems to be independent from the oxide material for I_CC_ > 20 μA [in agreement with the literature (Ielmini et al., 2012)], the LRS value shows a strong dependence on the oxide material for I_CC_ <20 μA. As expected, the largest oxide layer (HfO_2_/TaO_x_) exhibits the highest LRS values. Moreover, the LRS seems to depend strongly on I_CC_ in this low current range. Figure 8 represents the resistance variability σ_R_ of all tested oxide materials as a function of the mean resistance μ_R_. As we previously stated in Garbin et al. (2015), the LRS and HRS variabilities form a continuous curve and are thus presented together for each material. As one can see, σ_R_ increases with μ_R_, i.e., when I_CC_ is reduced. Indeed, the variability depends strongly on the resistance level but is identical for different oxide materials. The dependence of σ_R_ on μ_R_ is slightly reduced for $\mu_{\text{R}}>10^{6}\;\Omega$.

**Figure 9:**
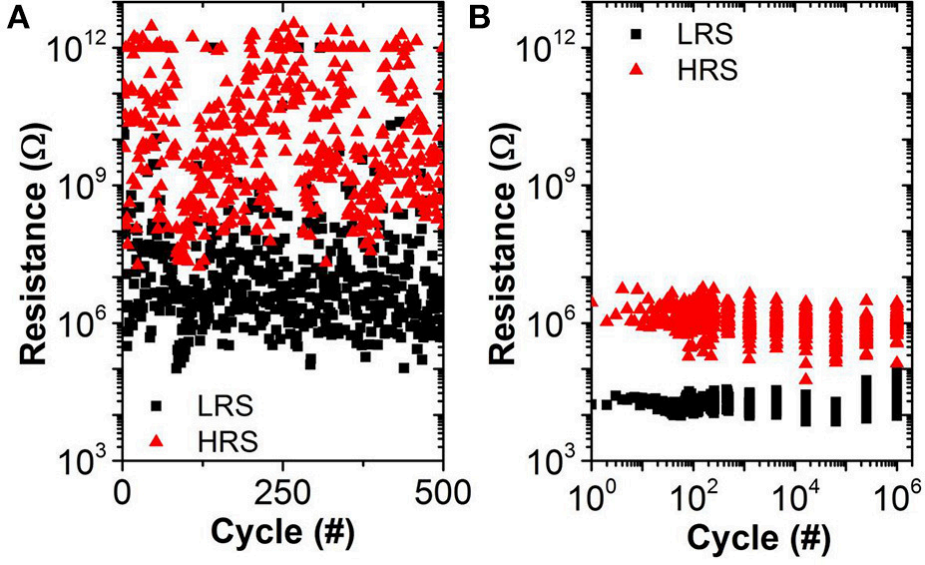
**TaO_**x**_/HfO_**2**_ endurance for pulsed operation using (A) I_**CC**_ = 5 μA, V_**Set**_ = 3 V, V_**Reset**_ = −1.5 V, t_**Set/Reset**_ = 10 μs (no resistance window) and (B) I_**CC**_ = 30 μA, V_**Set**_ = 2.5 V, V_**Reset**_ = −1.5 V, t_**Set/Reset**_ = 1 μs (1 decade median-median resistance window)**.

The experimental results (reduced I_Reset_, oxide dependent LRS, similar variability for LRS and HRS) may be explained by bulk switching and conduction mechanisms rather than filamentary ones (Chen et al., 2014; Goux et al., 2014) when very low I_CC_ (<20 μA) are used. We believe that in this case the current conduction in the LRS is dominated by trap-assisted tunneling as is the case for the HRS (Wong et al., 2012). This assumption is supported by experimental results from pulsed cycling of the OxRAM devices in both current regimes shown in Figure 9. Whereas I_CC_ = 30 μA is still sufficient to achieve a defined switching with a significant resistance margin between LRS and HRS (see Figure 9B), the LRS and HRS distributions for *I*_CC_ = 5 μA cover several orders of magnitude and are overlapping (i.e., no resistance window). In the case of *I*_CC_ > 30 μA, the resistance window can be improved by increasing the I_CC_.

**Figure 10:**
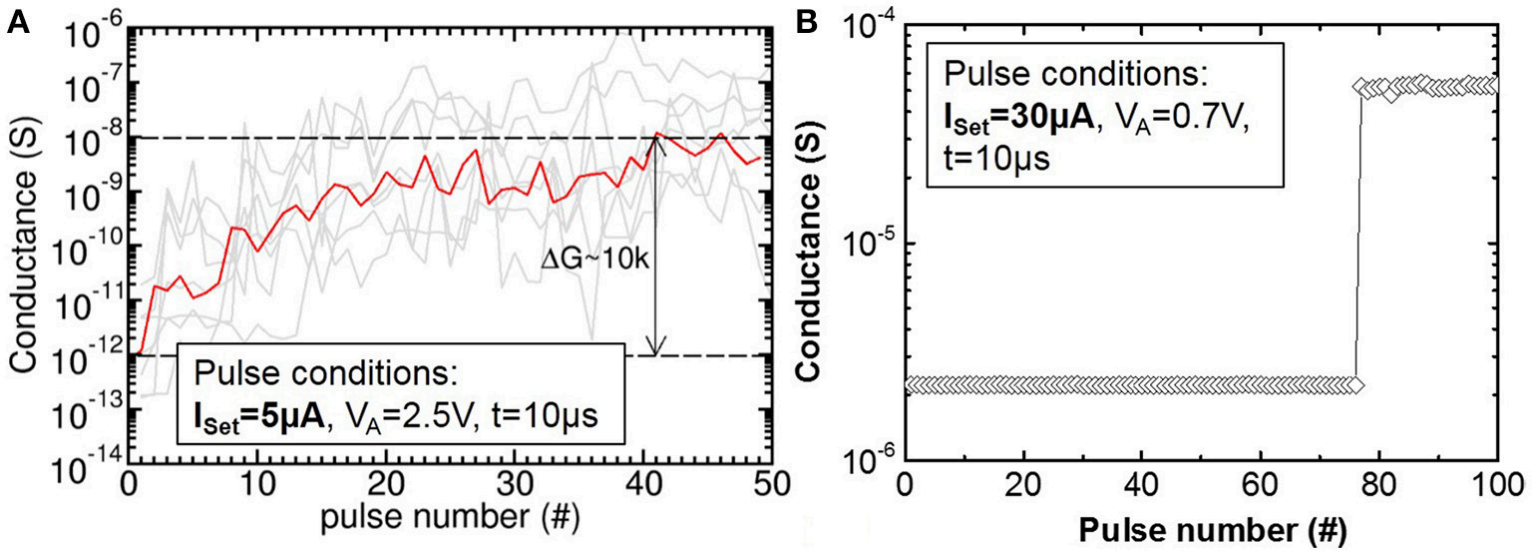
**(A)** Gradual Set (LTP) of TaO_x_/HfO_2_ devices (gray) obtained by application of 50 identical Set and Reset pulses and I_CC_ = 5 μA. Geometric mean for all devices (red). **(B)** Abrupt Set of TaO_x_/HfO_2_ for I_CC_ = 30 μA.

The dependence of the switching process on I_CC_ was experimentally studied in more detail by applying 50 identical Set or Reset pulses on the OxRAM device in HRS or LRS, respectively (see Figure 10). When a pulse with I_CC_ = 30 μA is repeatedly applied, the Set process occurs abruptly in a probabilistic manner after a few pulses and the achieved LRS does not change with more pulses (see Figure 10B). On the contrary, for pulses of I_CC_ = 5 μA, the Set process is no longer abrupt but rather progressive and the achieved LRS depends on the number of applied Set pulses (see Figure 10A). Note, that the conductance of single devices (gray lines) changes over several orders of magnitude orders with the pulse number while the different devices exhibit significant differences in conductance values (i.e., strong device-to-device variability).

## 5. OxRAM based synapses

Based on the electrical tests of OxRAM in the previous section, the HfO_2_/TaO_x_ resistive layer was chosen in this paper to implement the synapses since it has the highest resistance values compared to other tested materials (see Figure 7B) thus consuming the lowest power in read mode. Note, that the gradual resistance change observed in the ultra-low current OxRAM operation (using I_CC_ = 5 μA) seems promising for the implementation of LTP and LTD with one device per synapse, significantly reducing the circuit complexity [i.e., no pseudo-random number generator (PRNG) needed] and allowing for very compact low power synaptic networks. However, the device-to-device variability is in the same order of magnitude as the range of ΔG for single devices, thus preventing the gradual switching OxRAM based synapse from straightforward integration into a neuromorphic network circuitry. For this reason, the synapse implementation based on multiple abrupt switching OxRAM devices is adopted in this work. A number of OxRAM devices (*n*) operated in this manner (using I_CC_ = 30 μA) can be combined in a parallel architecture (Figure 5A) as described in detail in Garbin et al. (2015) to build one synapse featuring approximately *n* + 1 states of synaptic weight. 1T1R OxRAM structures have been fully characterized using a programming current I_CC_ = 30 μA and the experimental LRS and HRS distributions (from results in Figure 9B) have been integrated in the OxRAM based SNN architecture presented in Figure 4. Ten OxRAM devices were used per synapse resulting in a total number of 1600 OxRAM devices required for the SNN.

The stochastic STDP (see Section 3 for the description) can be achieved by using the intrinsic switching probability (tuning Set and Reset voltages) or by an extrinsic probability (tuning a pseudo random number generator, PRNG). In this work, the latter is used in combination with a driver circuit for the application of the Set and Reset electrical pulses with the corresponding probabilities (p_Set_ and p_Reset_). This allows to overcome the abrupt Set switching limitation of single OxRAM devices (Figures 9B, 10) inducing gradual/progressive Long Term Potentiation (LTP) and Long Term Depression (LTD) (Figure 5B).

## 6. Spike sorting performance of SNN application

**Figure 11:**
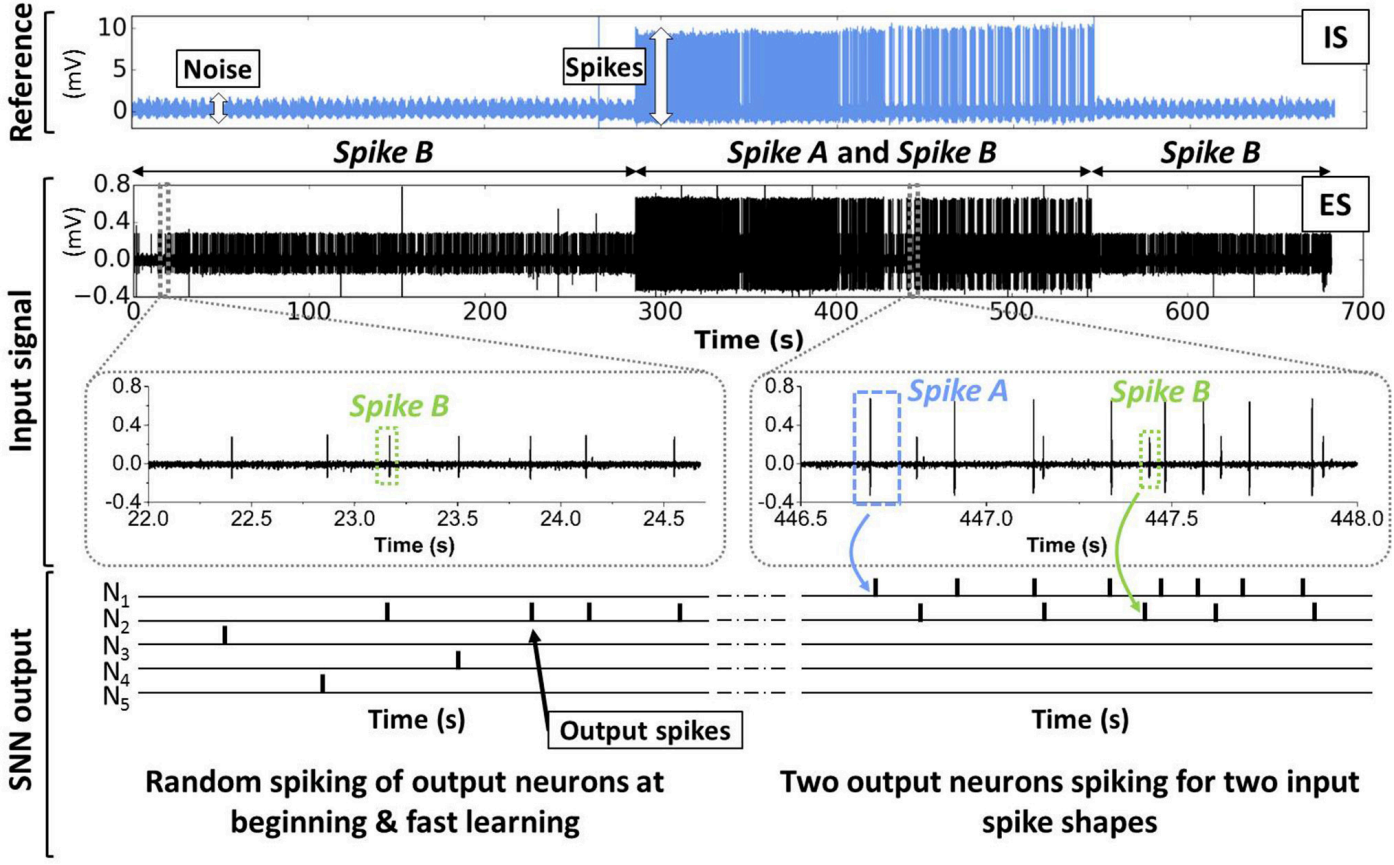
**Schematic illustration of the learning phase for the SNN (see Figure 4) applied on the biological data (see Figure 2)**. Initially, the SNN is untrained for new input spikes (in the ES signal) and output neurons spike randomly. Due to online learning, different output neurons become gradually selective to certain input spike patterns.

The complete spike sorting system consisting of band-pass filters and SNN was simulated with the “Xnet” (event-driven) simulator for the treatment of the Crayfish data (CF1) introduced in Section 2. Figure 11 illustrates schematically the unsupervised learning response of our SNN to the input signal (ES) described in Section 2. Initially (0 s–285 s), only Spike B is present in the ES. The SNN output, i.e. the firing patterns of the five output neurons *N*_1_–*N*_5_ are completely random. Thanks to the introduced lateral inhibition, one output neuron, here *N*_2_, becomes gradually selective to Spike B. Then (285 s–545 s), also Spike A is observed in the input signal. In this period, *N*_1_ starts to spike predominantly when the Spike A appears, while *N*_2_ continues to fire for Spike B. The remaining output neurons *N*_3_, *N*_4_, and *N*_5_ are rather silent. At the end of the test case (545 s–681 s) only Spike B is present. As expected, only *N*_2_ shows activity whereas *N*_1_, *N*_3_, *N*_4_, and *N*_5_ are inactive.

**Figure 12:**
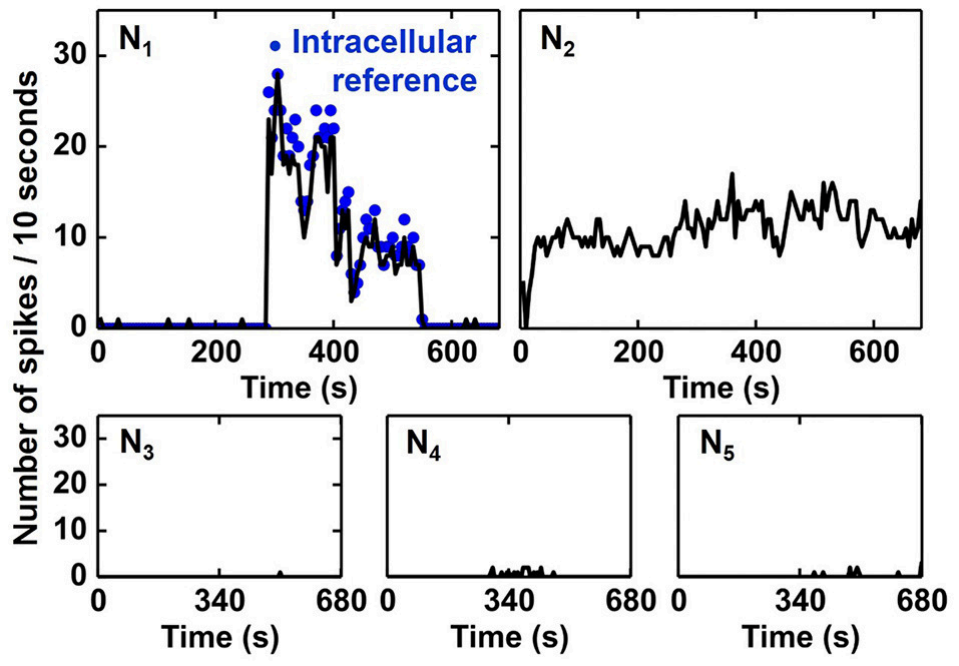
**Activity of SNN output neurons during 681 s of continuous input signal**. Activity is plotted as the number of spikes in time intervals of 10 s. *N*_1_ activity matches well with the intracellular reference (blue dots), i.e., *N*_1_ detects Spike A. *N*_2_ seems to be selective to Spike B, however, no reference data is available for verification.

The activities of all output neurons *N*_1_–*N*_5_ are shown in Figure 12 whereas the activity is defined as the number of output spikes in time intervals of 10 s. As one can see, the *N*_1_ activity is in good agreement with the intracellular reference, i.e. *N*_1_ detects Spike A. The activity of *N*_2_ is found to be correlated to Spike B, however, no ground truth (intracellular signal) is available for a reliable quantification of the recognition rate. *N*_3_, *N*_4_, and *N*_5_ show very small activity meaning that they do not become selective to input spikes in the ES. These results prove the qualitative functionality of the proposed spike sorting algorithm. Note that, even if the frequency patterns of Spike A and B are overlapping, two independent output neurons are assigned for the two different spikes.

**Figure 13:**
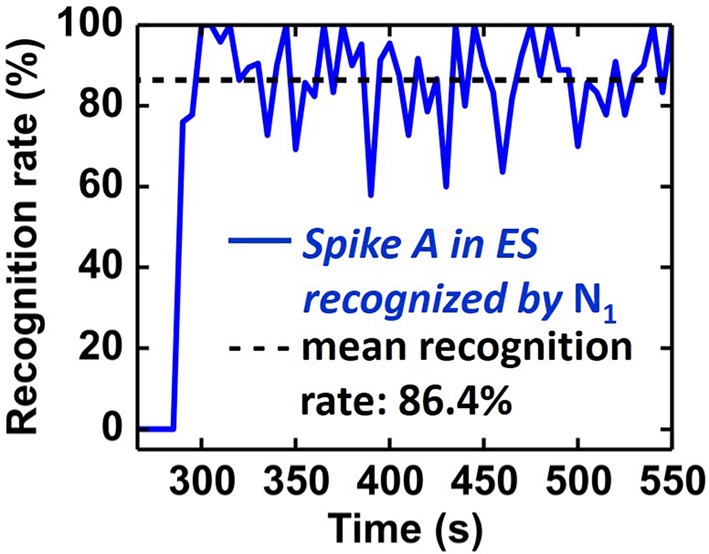
**Temporal evolution of recognition rate of Spike A by ***N***_**1**_**. A mean recognition rate of 86.4% (dashed line) is reached within 15 s starting from the first Spike A occurence.

In order to quantify the recognition rate of Spike A (Figure 12), we correlated the activity of *N*_1_ with the intracellular signal (IS in Figure 11). A Spike A event is considered to be recognised by *N*_1_ if *N*_1_ spikes within 20 ms after the Spike A event. The recognition rate was calculated as the ratio of recognized spikes to the total number of Spike A events (truth from IS data) in a given time interval (fixed to ten seconds). As shown in Figure 13, the system reached its mean spike recognition rate of 85.5% after 15 s (corresponding to 50 Spike A events), calculated starting from the first occurrence of Spike A in the ES signal at (*t* = 285 s).

Table 2 summarizes the statistics of the SNN for the application on the ES data used in this work. The total duration of the signal is 681 s and the activity of all neuronal and synaptic events was recorded. Note, that the average number of set and reset events per OxRAM device is very small, 17 and 37, respectively. This means that the SNN learning is fast and stable and OxRAM device degradation can be neglected. Furthermore, extrapolation of these statistics to an application time of 10 years, accounts to 8 × 10^6^ Set and 1.7 × 10^7^ Reset events per OxRAM device. Note, that these cycling requirements are satisfied by state-of-the-art OxRAM technologies (Garbin et al., 2015).

**Table 2:** **SNN statistics**.

**Input signal duration**	**681 s**
Number of synapses	160
Devices/synapse	10
Read events	16.2 × 10^6^
Set events	27.5 × 10^3^
Reset events	58.6 × 10^3^
Number of spikes	330 × 10^3^

We estimated the specific energy dissipation for a single synaptic event in our SNN by considering the pre-defined operation conditions for the OxRAM devices according to:

(1) $$\begin{array}{l} {\text{E}}_{\text{mode}}={\text{V}}_{\text{mode}}\;\cdot \;{\text{I}}_{\text{mode}}\;\cdot \;{\text{t}}_{\text{mode}} \end{array}$$

where the index mode = [Set, Reset, Read] denotes the type of synaptic event. V_mode_, I_mode_, and t_mode_ are the respective values for the voltage, current, and time of the applied pulse. For Set and Reset, the pulse conditions reported in Figure 9B were used. For the Read operation, V_Read_ = 0.1V and t_Read_ = 1μs whereas I_Read_ is determined by the device resistance. Based on the statistics reported in Table 2 and the event specific energies, the total energy dissipation and corresponding power consumption P = E/t of the synaptic part of the SNN are calculated following to:

(2) $$\begin{array}{l} {\text{E}}_{\text{total}}=\underset{\text{mode}}{\sum }{\text{E}}_{\text{mode}}\;\cdot \;{\text{N}}_{\text{mode}} \end{array}$$

whereas N_mode_ is the number of Set, Reset, or Read events. The estimated energy consumptions of the synaptic part of the SNN are reported in Table 3. The event specific energies in the low pJ range in combination with the relatively low number of switching events, result in extremely low synaptic power consumption of 8.1 nW. Considering a state-of-the-art analog neuron design in the 65 nm technology node (Joubert et al., 2012) with an energy per spike of 2 pJ may add 0.66 μJ (i.e., 5.6%) to the total energy dissipation. Hence, the power consumption remains at a very low competitive level of 8.6 nW.

**Table 3:** **SNN power metrics**.

**Energies per event**	
Set event (E_Set_)	75 pJ
Reset event (E_Reset_)	45 pJ
Read event (E_Read_)	0.39 pJ
**Total power estimation**	
Energy dissipation	11 μJ
Power consumption	8.1 nW

**Figure 14:**
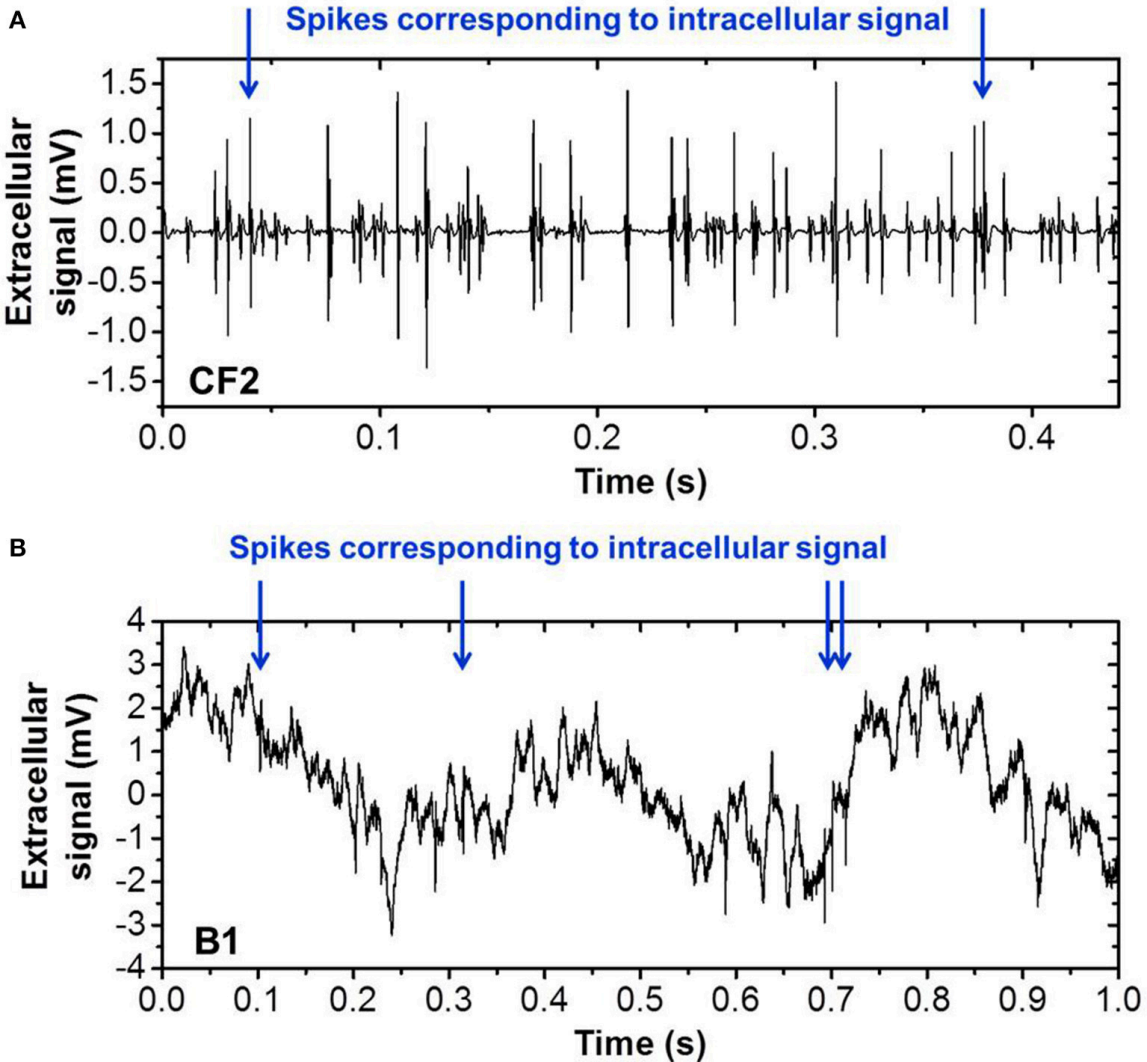
**Sequences of real biological spiking data used for verification of Spike Sorting system, recorded in (A) ***in-vitro*** crayfish (Cattaert and Manira, 1999) and (B) ***in-vivo*** implanted rat hippocampus (Henze et al., 2000)**. Intracellular recordings were simultaneously obtained and provide the ground truth for valid quantification of the spike recognition rate for the labeled spikes (blue arrows).

We tested our spike sorting SNN with respect to its applicability on other neural spiking data. Therefore, we used another dataset recorded (*in-vitro*) from Crayfish and a dataset recorded from anesthetized (*in-vivo*) rat hippocampus [publicly available online provided by the Buszaki lab (Harris et al., 2000; Henze et al., 2000)]. Both datasets feature simultaneous recording of extra- and intra-cellular signals and are in the following referred to as CF2 and B1, respectively. As before in the case of CF1, we use the intracellular recording as a ground truth for the quantification of the recognition rate of the SNN output. CF2 is more complex with respect to CF1 since it contains more different spike shapes and a higher overall spiking frequency which results in overlapping spikes. B1 comprises a strongly increased background noise level with respect to CF1. Snapshots of both datasets are shown in Figure 14. Without changing the parameters of our filter bank and SNN, the recognition rate for CF2 is 74.2 and 82.1% for B1. These results confirm that thanks to the STDP learning rule, the proposed network can be used on different biological data without tuning parameters. State-of-the-art spike sorting algorithms based on spike detection, feature extraction, and clustering (i.e., standard methodology) achieve recognition rates around 90% on the dataset B1 (Gasthaus and Wood, 2008; Gasthaus et al., 2008) and therefore outperform our proposed approach slightly in terms of accuracy. However, the reported method does not incorporate a spike detection step but uses previously extracted and aligned spike waveforms for the classification. Moreover, the mathematical algorithm is rather complex. For this reason, the standard approach seems impractical for real-time applications with low power consumption (for portability).

Finally, we make a qualitative comparison between our SNN-based spike sorting approach with the standard (template matching, PCA) methodologies in Table 4. The advantage of our approach is clearly the real-time functionality without the need for supervision as well as the computational efficiency which results in very low power consumption. These benefits may enable our approach to be suitable for rather simple hardware implementation for long-time, portable, and low-power implants whereas standard spike sorting techniques do not meet these requirements. On the other hand, the spike sorting accuracy is lower with respect to standard techniques. This issue might be addressed by a more sophisticated network (e.g., more neuron layers, better data encoding etc.).

**Table 4:** **Qualitative comparison of Spike-Timing Depending Plasticity (STDP) based spike sorting (this work) with standard approaches (template matching, PCA)**.

**Criterion**	**STDP based (this work)**	**Standard techniques**
Real-time functionality	+	−
(permanent adaptation		
to spikes shapes)		
Unsupervised operation	+	−
Computational efficiency	+	−
Energy efficiency	+	−
Accuracy	−	+
Suitability for (long-term)	+	−
hardware integration		

## 7. Conclusion

In this paper, we demonstrated the high potential of possible hardware embedded Spiking Neural Networks (SNN) for spike sorting of brain activity signals, relevant for the analysis of large-scale brain signals. We showed that these systems allow for fast adaptation to new input data and completely unsupervised operation, independently from the number of spikes in the input signal. The network has been tested on different sets of real biological spiking data and functionality was proven for all datasets without parameter tuning. In contrast to standard spike sorting techniques, SNN based approaches offer several advantages, e.g., no power-consuming CPU or GPU are needed and no parameters (e.g., threshold level for spike detection) have to be optimized manually as a function of the input data. Hence, SNN's offer a powerful alternative to standard spike sorting methodologies. We proposed OxRAM technology for the hardware implementation of synapses with ultra-low power consumption and fast operation times (<1μs). This enables the system for real-time application to neural data in potential medical devices featuring high energy-efficiencies. Moreover, extended OxRAM cycling capabilities (>10^8^ switching cycles) allow for long-term functional implants. Spike sorting performances are lower with respect to conventional power-hungry spike sorting methodologies and may be improved by more sophisticated SNN designs and/or complementary input information. Nevertheless, thanks to the unsupervised real-time functionality and low-power hardware compatibility, we believe that compact hardware implementations of SNN's will enable spike sorting directly at the recording site within the brain thus solving the bottleneck of data storage and power consumption. Furthermore, data reduction rates of about 1000 (depending on the spiking frequency of the input data) open the path to wireless data streaming of the spike sorted data to an external receiver.

## Author contributions

TW provided simulations of the neural network and contributed to experimental data. OB and DG contributed to the definition and optimization of the neural network. DC provided the biological data. All authors discussed the results and contributed to manuscript preparation. EV and BY supervised the research.

### Conflict of interest statement

The authors declare that the research was conducted in the absence of any commercial or financial relationships that could be construed as a potential conflict of interest.
