# Corrigendum: Spiking Neural Networks Based on OxRAM Synapses for Real-Time Unsupervised Spike Sorting

**DOI:** 10.3389/fnins.2017.00486

**Published:** 2017-08-29

**Authors:** Thilo Werner, Elisa Vianello, Olivier Bichler, Daniele Garbin, Daniel Cattaert, Blaise Yvert, Barbara De Salvo, Luca Perniola

**Affiliations:** ^1^Laboratoire d'Électronique et de Technologie de l'Information (LETI), Commissariat à l'Énergie Atomique et aux Énergies Alternatives (CEA) Grenoble, France; ^2^Université Grenoble Alpes Grenoble, France; ^3^Laboratoire d'Intégration de Systèmes et de Technologies (LIST), Commissariat à l'Énergie Atomique et aux Énergies Alternatives (CEA) Gif-sur-Yvette, France; ^4^Institut de Neurosciences Cognitives et Intégratives d'Aquitaine, Université de Bordeaux, CNRS Bordeaux, France; ^5^BrainTech Laboratory U1205, Institut National de la Santé et de la Recherche Médicale Grenoble, France; ^6^BrainTech Laboratory U1205, Université Grenoble Alpes Grenoble, France

**Keywords:** brain-computer interfaces, neuromorphic computing, OxRAM, resistive RAM (RRAM) synapse, spike sorting, spiking neural network, spike timing-dependent plasticity

The way we presented the results in the original article may suggest that the proposed spike-sorting approach managed to achieve an accuracy of 90% classification, while, as it can be inferred from the study, this referred to a detection rate not accounting for false positives.

We would thus like to make the results clearer by modifying the text as follows:

The end of the Abstract should read: This artificial SNN is able to identify, learn, recognize and distinguish between different spike shapes in the input signal without any supervision.

The end of the second paragraph of the “Spike Sorting Performance of SNN Application” Section on page 9 should read: As shown in Figure 13, the system reached its mean spike recognition rate of 85.5% after 15 s (corresponding to 50 Spike A events), calculated starting from the first occurrence of Spike A in the ES signal at (*t* = 285 s), with a false positive rate of 6.9%.

The “Spike Sorting Performance of SNN Application” Section paragraph at the beginning of page 10 should read: Without changing the parameters of our filter bank and SNN, the recognition rate for CF2 is 74.2 and 82.1% for B1. This still high detection rate was however accompanied by a poorer classification accuracy with a high number of false positives (274% for CF2 comprising many overlapping waveforms and 61% for B1 displaying a lower signal-to-noise ratio, as compared to 6.9% for CF1), suggesting that further efforts remain to be put to improve the proposed approach to make it robust in all cases.

## Conflict of interest statement

The authors declare that the research was conducted in the absence of any commercial or financial relationships that could be construed as a potential conflict of interest.

